# Multi-drug resistant non-typhoidal *Salmonella* associated with invasive disease in western Kenya

**DOI:** 10.1371/journal.pntd.0006156

**Published:** 2018-01-12

**Authors:** Adam Akullian, Joel M. Montgomery, Grace John-Stewart, Samuel I. Miller, Hillary S. Hayden, Matthew C. Radey, Kyle R. Hager, Jennifer R. Verani, John Benjamin Ochieng, Jane Juma, Jim Katieno, Barry Fields, Godfrey Bigogo, Allan Audi, Judd Walson

**Affiliations:** 1 Institute for Disease Modeling, Global Good Fund, Bellevue, Washington, United States of America; 2 Department of Global Health, University of Washington, Seattle, WA, United States of America; 3 Division of Global Health Protection, Center for Global Health, Centers for Disease Control and Prevention, Atlanta, GA, United States of America; 4 Department of Microbiology, University of Washington, Seattle, WA, United States of America; 5 Kenya Medical Research Institute/CDC Research and Public Health Collaboration, Kisumu, Kenya; 6 Division of Global Health Protection, Center for Global Health, Centers for Disease Control and Prevention Kenya, Nairobi, Kenya; 7 Childhood Acute Illness and Nutrition Network, Nairobi, Kenya; Liverpool School of Tropical Medicine, UNITED KINGDOM

## Abstract

Non-typhoidal *Salmonella* (NTS) is a leading cause of bloodstream infections in Africa, but the various contributions of host susceptibility versus unique pathogen virulence factors are unclear. We used data from a population-based surveillance platform (population ~25,000) between 2007–2014 and NTS genome-sequencing to compare host and pathogen-specific factors between individuals presenting with NTS bacteremia and those presenting with NTS diarrhea. *Salmonella* Typhimurium ST313 and *Salmonella* Enteritidis ST11 were the most common isolates. Multi-drug resistant strains of NTS were more commonly isolated from patients presenting with NTS bacteremia compared to NTS diarrhea. This relationship was observed in patients under age five [aOR = 15.16, 95% CI (2.84–81.05), P = 0.001], in patients five years and older, [aOR = 6.70 95% CI (2.25–19.89), P = 0.001], in HIV-uninfected patients, [aOR = 21.61, 95% CI (2.53–185.0), P = 0.005], and in patients infected with *Salmonella* serogroup B [aOR = 5.96, 95% CI (2.28–15.56), P < 0.001] and serogroup D [aOR = 14.15, 95% CI (1.10–182.7), P = 0.042]. Thus, multi-drug-resistant NTS was strongly associated with bacteremia compared to diarrhea among children and adults. This association was seen in HIV-uninfected individuals infected with either *S*. Typhimurium or *S*. Enteritidis. Risk of developing bacteremia from NTS infection may be driven by virulence properties of the *Salmonella* pathogen.

## Author summary

Though NTS is normally associated with self-limiting gastroenteritis in humans, it is a leading cause of bloodstream infection in Africa. The biological mechanisms that contribute to invasiveness in NTS in Africa are unclear. In this paper we address which specific host and pathogen risk factors are associated with blood stream infection from non-typhoidal *Salmonella* in rural Kenya. We found that multi-drug resistant (MDR) strains of NTS were associated with NTS bacteremia, even after controlling for known host-factors including HIV, age, and NTS serogroup (a taxonomic grouping). Our results suggest that multi-drug resistant NTS is associated with blood stream infection even in the immune-competent host. *Salmonella* Typhimurium sequence type ST313, an emerging genotype in sub-Saharan Africa, was the most common cause of blood stream infection in children and adults, followed by *Salmonella* Enteritidis sequence type ST11. The increasing prevalence of commonly circulating non-typhoidal *Salmonella* poses a major challenge to the control of highly pathogenic NTS serovars. The specific biological and epidemiological mechanisms driving invasiveness from infection with drug-resistant NTS warrant further investigation.

## Introduction

Non-typhoidal *Salmonella* (NTS) is a leading cause of bacteremia in Africa [1]. Whereas NTS commonly manifests as self-limiting diarrhea in immune-competent individuals, NTS bacteremia occurs at high rates in young children (175–388 cases per 100,000) [2, 3] and HIV-infected adults (2000–7500 cases per 100,000) [4–8] in sub-Saharan Africa and may also be associated with malaria co-infection and malnutrition in children [9–14].

In addition to its association with certain host-factors and co-infections, recent evidence suggests that NTS bacteremia in Africa may be associated with the emergence of novel *Salmonella* Typhimurium genotypes that have undergone considerable genomic reduction, which may suggest human adaptation leading to invasive blood-stream infection [15–24]. The recent emergence of multi-drug resistance (MDR) (defined as resistant to trimenthroprim-sulfamethoxasol [TMP-SMX], ampicillin, and chloramphenicol) has also been observed in dominant serovars of *Salmonella* in Africa, including *S*. Typhimurium and *S*. Enteritidis [16, 17, 19, 25–28].

Additionally, a clone of *S*. Typhimurium (ST313), which has undergone considerable genomic reduction, is a common cause of bacteremia in HIV infected individuals and children across sub-Saharan Africa [16]. Since invasive NTS infections frequently occur in individuals with significant co-morbidities, including HIV infection, malaria, and malnutrition, it remains unclear whether genome reduction, and associated changes in drug resistance and virulence, has contributed to high rates of NTS bacteremia in Africa [16, 17, 24, 29].

Here we test for associations between NTS bacteremia and both host and pathogen-specific factors. Using a large population-based surveillance cohort from rural Kenya, we compared individuals with NTS bacteremia to those with NTS diarrhea to elucidate host and pathogen-related risk factors for NTS invasiveness. Finally, we genotyped a subset of NTS isolates to determine the phylogenetic lineages contributing to the high burden of NTS bacteremia in western Kenya.

## Methods

### Ethics statement

This study was nested within an ongoing population-based infectious disease surveillance system (PBIDS) conducted since 2006 by the Kenya Medical Research Institute (KEMRI) in collaboration with the US Centers for Disease Control and Prevention (CDC), and the methods have been described previously [28, 30, 31]. The PBIDS protocol was reviewed and approved by the Institutional Review Boards of KEMRI and CDC. All adult subjects provided written, informed consent and a parent or guardian of any child provided informed consent on their behalf.

### Study site

The surveillance cohort consists of approximately 25,000 individuals of all ages residing in Asembo, a rural region of western Kenya located in Siaya County on Lake Victoria. The region is characterized by low population density, intense, year-round malaria transmission and an adult HIV prevalence of 15–17% [28].

Inclusion into the surveillance required the following: 1) currently reside within a village whose epicenter is located no more than 5 kilometers from the Lwak Mission Hospital (LMH) in Asembo, 2) have resided permanently in the area for at least four calendar months, and 3) have provided written informed consent/assent. PBIDS participants are visited biweekly in their households and asked about acute illnesses, healthcare seeking, and changes to the household (i.e. births, deaths, migrations). Participants can access free health care for acute illnesses at LMH.

### NTS bacteremia and diarrhea

At LMH, blood samples for culture are collected from patients meeting the case definition for 1) severe acute respiratory infection (in children <5 years cough or difficulty breathing, plus lower chest indrawing or danger sign [unable to drink/feed, vomiting everything, convulsions, lethargy, unconscious, stridor] or oxygen saturation <90%; in those ≥5 years cough or difficulty breathing or chest pain plus temperature ≥ 38.0 degrees C or oxygen saturation <90%), 2) acute febrile illness (temperature ≥ 38.0 degrees C), 3) jaundice, or 4) hospital admission (whether or not fever was present). Due to the large number of individuals with febrile illnesses reporting to Lwak Mission Hospital, only the first two individuals over/under age five years presenting with acute febrile illness each day had blood collected for culture; there were no limits on blood culture collected for severe acute respiratory infection, jaundice or hospitalization.

### Malnutrition

Height and weight were measured at time of clinical presentation for children under five years of age. Height-for-age (HAZ) and weight-for-height z-score (WHZ) cut-offs of -2.0 were used to classify stunting and wasting, respectively, in children under five years of age based on 2006 World Health Organization child growth standards [32]. Z-scores were excluded if HAZ was below -6 or above 6, WAZ below -6 or above 5, WHZ below -5 or above 5 [32].

### HIV

HIV status was ascertained on a subset of NTS patients through two HIV home-based counselling and testing campaigns (2008/2009 and 2013) conducted on all consenting individuals ≥ age 13 years present in the disease surveillance cohort at the time of the survey. Children under 13 years of age were only tested if their parent was HIV infected. HIV data from these two community-level surveys were then linked to the clinic database through patient records. Analysis was done on the full subset of individuals linked to an HIV status, assuming that individuals who tested positive for HIV after NTS diagnosis were HIV infected at NTS diagnosis and those who tested negative for HIV prior to NTS diagnosis were HIV uninfected at NTS diagnosis. Where sample size allowed we secondarily restricted our analysis to those whose status could be confirmed at NTS diagnosis, (i.e., tested positive before NTS diagnosis, tested negative after NTS diagnosis, or children under 13 tested at any time prior to NTS diagnosis).

### Current malaria parasitemia

Blood smears for malaria diagnosis were obtained from all individuals presenting with acute febrile illness and examined by trained microscopists on site. Malaria at the time of NTS diagnosis was defined as the presence of asexual stages of *Plasmodium falciparum* on microscopy using thick smear collected on the same day as stool/blood culture yielding NTS.

### Bacterial culture and identification

Laboratory methods for culturing bacterial pathogens from blood have been described previously [28]. Briefly, blood (7–10 ml for adults and 1–3 ml for children) was inoculated into BACTEC culture bottles (Becton, Dickinson and company, Sparks, MD, USA) and incubated in an automated BACTEC 9050 at 35°C for 1–5 days. A Gram-stained smear was prepared from any bottle with growth, and the broth sub-cultured onto standard enriched culture media for further identification. Stool specimens were processed according to standard protocols described elsewhere [33]. Colonies of *Salmonella* were identified and confirmed using an API 20E system (Appareils et Procedes d’Identification, Montalieu Vercieu, France) following manufacturers’ instructions. Commercial agglutinating antiserum (Denka Seiken, Tokyo, Japan) was used to serotype *Salmonella* isolates (Krieg NR, Holt JG (1984) Bergey’s Manual of Systematic Bacteriology. Baltimore: Williams and Wilkins. pp 427–58)

### Antimicrobial susceptibility

Antimicrobial susceptibility patterns were determined using standard Kirby-Bauer disc diffusion techniques after incubation for 16 hours [34]. Susceptibility was classified according to three MIC cutoff values (resistant, intermediate, and susceptible) for NTS as defined by Clinical and Laboratory Standards Institute CLSI [34]. Multi-drug resistance (MDR) was defined as resistance to chloramphenicol, trimethroprim-sulfamethoxasol (TMP-SMX), and ampicillin [28]. For purposes of analysis, NTS isolates were classified as resistant or non-resistant whereby non-resistant included susceptible and intermediate resistance.

### Whole genome sequencing

NTS isolates obtained from a subset of individuals who provided consent after sample collection were sent to laboratories at the University of Washington for whole genome sequencing to assess the phylogenetic relatedness of the bacteremic and diarrheal NTS isolates in western Kenya. To isolate genomic DNA for sequencing, strains were grown overnight at 37°C with shaking in 3 mL of Luria Broth (BD Biosciences, USA). Genomic DNA was isolated using Gentra Puregene Yeast/Bact. Kit (Qiagen, Valencia, CA), according to manufacturer’s directions. For each genome standard Illumina Nextera or Nextera XT libraries were constructed according to manufacturer’s guidelines (Illumina Inc., San Diego, CA). Prior to the Nextera XT library normalization/denaturation step, double stranded libraries were normalized with the Invitrogen SequalPrep Normalization Plate Kit (Thermo Fisher Scientific/Life Technologies, Grand Island, NY). Paired-end libraries for each genome were used to generate 100 bp or 150 bp reads with the Illumina HiSeq 2000 or MiSeq. Sequencing of libraries was performed according to manufacturer’s standards (Illumina Inc., San Diego, CA).

### Multilocus sequence typing (MLST)

Sequence types (STs) were generated from whole genome shotgun (WGS) sequence reads using the MLST scheme developed by Achtman et al. 2012 [35], which uses fragments from seven housekeeping genes: aroC, dnaN, hemD, hisD, purE, sucA and thrA. We created a single MLST reference sequence comprised of the scheme’s seven allele template. The Burrows-Wheeler Alignment (BWA) algorithm [36] was used to align sequence reads from each strain to the MLST reference sequence using an edit distance of 10, which allowed alignment of reads with up to 10 mismatches. Custom scripts were used to parse alignments to produce consensus sequences for each housekeeping gene, compare consensus sequences to the MLST allele database at EnteroBase (http://enterobase.warwick.ac.uk), and generate STs based on the MLST database of 7 allele combinations.

### Phylogenetic analysis of *S*. *Typhimurium*

Phylogenetic analysis was restricted to *S*. *Typhimurium* isolates and was generated from whole genome sequences using the kSNP software package version 2.1.1., in which SNP discovery was based on k-mer analysis, i.e. single variant positions within sequences of nucleotide length k [37]. The maximum likelihood tree was constructed using 31-mers that were identified in at least 50% of the strains and was based on 3,058 SNPs. The 50% requirement provided phylogenetic resolution of the *S*. Typhimurium strains while excluding the SNPs present in only one or a small number of genomes, which are more likely to be the result of sequencing or assembly errors or from mobile elements such as phages or plasmids. Support for branch nodes was computed by FastTree version 2.1.3 [38], which is provided in the kSNP package. Tree branches are expressed in terms of changes per total number of SNPs, not changes per site, as SNP-based trees do not include invariant sites. Local support values are based on the Shimodaira-Hasegawa test on the three alternate topologies at each split in the tree. The tree was drawn using Dendroscope version 3.2.10 [39].

### Case definition

NTS bacteremia cases were defined as those with at least one NTS serotype isolated from the blood. Stool samples were collected from patients presenting with diarrhea (≥3 looser than normal stools in a 24-hour period), and those with NTS isolated from the stool, (and did not meet indication for blood culture or were blood culture negative), were classified in this analysis as NTS diarrheal cases. Any individuals with NTS isolated from both blood and stool culture were classified as NTS bacteremia cases. Repeat visits within one month of the original NTS diagnosis in which NTS was isolated from either blood or stool were excluded to avoid counting the same episode of NTS twice.

### Statistical analysis

Logistic regression with robust standard errors was used to compare the odds of host and pathogen characteristic between NTS bacteremia and NTS diarrhea cases in univariate and multivariate models. Multivariate models were stratified on age over and under five years and were adjusted for potential confounders defined *a priori*, including age (continuous), year of diagnosis, malaria smear positivity, and multi-drug resistant phenotype. Because HIV is a major risk factor for NTS bacteremia, adjusted odds ratios were further stratified on HIV status to estimate the independent effects of pathogen-specific factors on clinical outcome in both HIV-infected and HIV-uninfected groups.

## Results

### Study population

**Fig 1:**
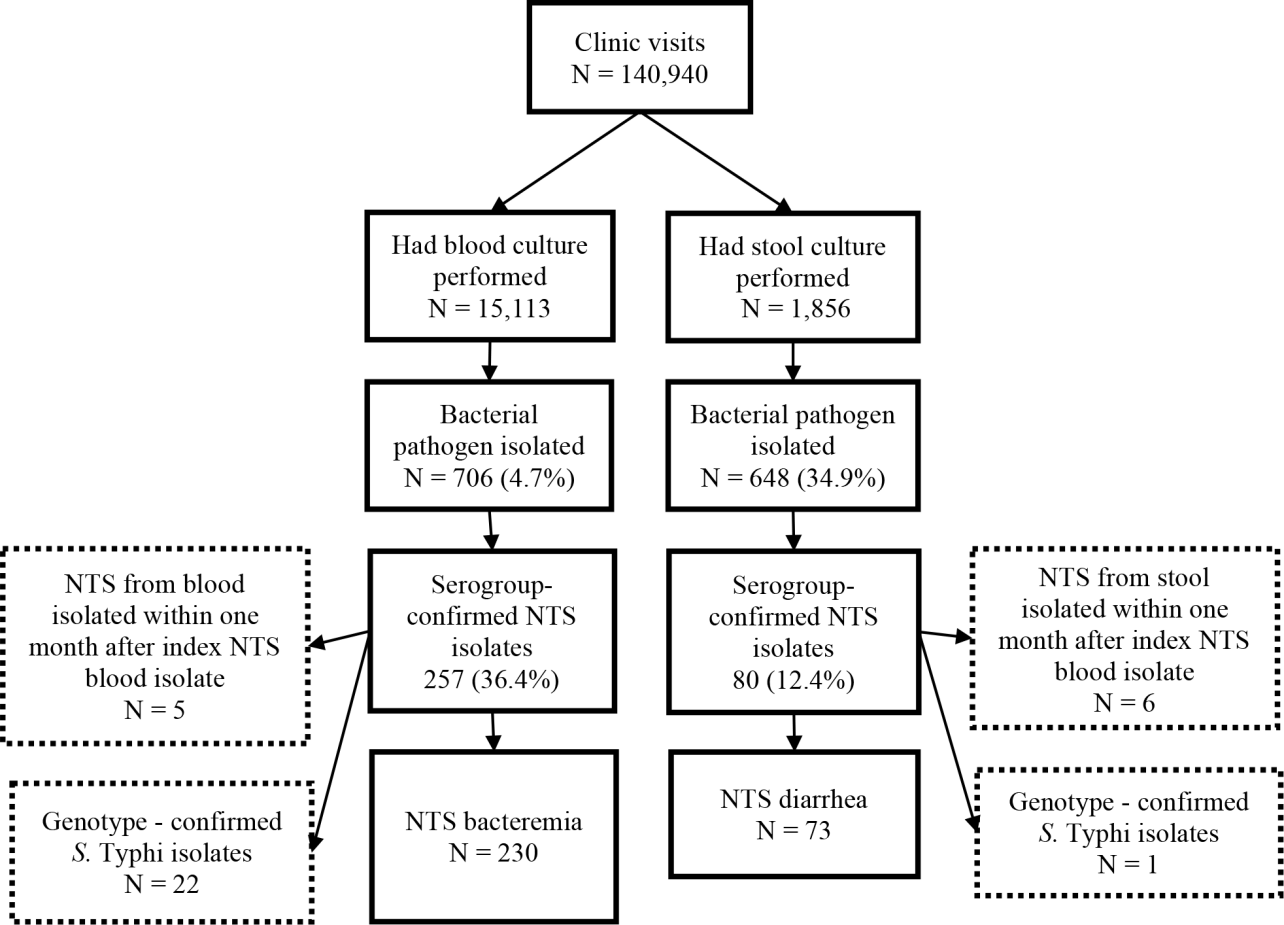
Sample flow chart for inclusion of NTS clinical isolates.

Between January 1, 2007 and December 31, 2014 there were 140,940 clinic visits to Lwak Mission Hospital among 24,748 unique individuals (Fig 1). Among 15,113 individuals who underwent blood culture, 842 had at least one bacterial pathogen isolated of which 136 were identified as contaminants, resulting in 706 (4.7%) bacterial pathogens isolated, of which 257 (36.4%) were NTS. Among 1,856 individuals whose stool specimens were tested by culture, 648 (34.9%) grew one or more bacterial pathogens, of which 80 (12.4%) were NTS.

Among the 257 blood NTS isolates, 230 met the inclusion criteria for NTS bacteremia. Five blood NTS isolates were excluded as they were secondary blood culture NTS isolates occurring within one month of the index blood culture NTS episode. Twenty-two blood culture NTS isolates were excluded after being identified as *S*. Typhi through subsequent full genome sequencing of a subset of NTS isolates. Among 80 NTS isolates from stool, 73 met the criteria for diarrheal NTS. Among stool culture positive NTS isolates, 6 were excluded due to their occurrence in the same individual within one month of an index case of either blood or stool culture positive NTS. One stool NTS isolate was excluded after being identified as *S*. Typhi through subsequent full genome sequencing.

Among the 230 NTS bacteremia cases included in the analysis, 59 (25.7%) were linked to an HIV status ascertained from either of two population-based HIV surveys conducted by CDC-Kenya in 2008/2009 and 2013. Among 73 NTS diarrhea cases, 28 (38.4%) were linked to an HIV status from the same surveys.

Comparison of demographic, clinical, and pathogen-specific factors between NTS bacteremia and NTS diarrhea cases.

Among children under age five, those with NTS bacteremia tended to be older (mean age 2.2 years) than those with NTS diarrhea (mean age 1.3 years), P = 0.003 (Table 1). Among cases age five and older, those with NTS bacteremia tended to be younger (mean age of 26.9 years) than those with NTS diarrhea (mean age of 32.5 years), though the difference was not significant. Among cases 5 years and older with any HIV testing result, HIV prevalence was higher among bacteremia cases compared with diarrheal cases (71.7% versus 40.7%, P = 0.018). This difference held when restricted to those whose HIV status was known at the time of NTS infection (79.2% versus 16.7%, P = 0.009). Co-infection with malaria was more common in bacteremic versus diarrheal patients in both age-groups, though the difference was not significant.

**Table 1:** Prevalence of host- and pathogen- characteristics by NTS clinical outcome (bacteremia versus diarrhea), stratified on age under and over 5 years.

		Age < 5 years			Age ≥5 years	
	NTS diarrhea	NTS bacteremia	P—value^1^	NTS diarrhea	NTS bacteremia	P—value^1^
	n = 16	n = 117		n = 57	n = 113	
	n (%) / mean (sd)	n (%) / mean (sd)		n (%) / mean (sd)	n (%) / mean (sd)	
Host factors						
Age (mean (sd))	1.32 (0.90)	2.22 (1.14)	**0.003**	32.46 (20.19)	26.87 (18.13)	0.070
Sex (% female)	5 (31.2)	57 (48.7)	0.285	30 (52.6)	68 (60.2)	0.438
Nutritional status at presentation						
Wasted (WHZ < -2.0)	3 (21.4)	16 (14.8)	0.456	NA	NA	NA
Stunted (HAZ < -2.0)	6 (40.0)	33 (29.7)	0.610	NA	NA	NA
Severe acute malnutrition (WHZ < -3.0)	1 (7.1)	7 (6.5)	1.000	NA	NA	NA
Any medication taken for illness prior to presentation^2^	7 (43.8)	64 (54.7)	0.578	29 (50.9)	52 (46.0)	0.663
TMP/SMX taken	1 (6.2)	9 (8.0)	1.000	2 (3.8)	3 (2.9)	1.000
Other antibiotic taken	0 (0.0)	2 (1.8)	1.000	5 (9.6)	2 (1.9)	**0.043**
Received HIV test	1 (6.3)	13 (11.1)	0.552	27 (47.4)	46 (40.7)	0.408
HIV positive^3^	0 (0.0)	6 (46.2)	1.000	11 (40.7)	33 (71.7)	**0.018**
HIV positive (status confirmed at NTS dx)	0 (0.0)	6 (46.2)	1.000	1 (16.7)	19 (79.2)	**0.009**
Malaria smear positive^4^	3 (21.4)	33 (29.2)	0.756	3 (7.3)	19 (19.6)	0.080
Pathogen factors						
Serogroup ^5^			**<0.001**			**<0.001**
Group B	7 (43.8)	75 (64.1)		28 (49.1)	77 (68.8)	
Group C1/C2	4 (25.0)	0 (0.0)		13 (22.8)	0 (0.0)	
Group D	3 (18.8)	40 (34.2)		11 (19.3)	35 (31.2)	
Undefined	2 (12.5)	2 (1.7)		5 (8.8)	0 (0.0)	
*Salmonella* sequence type (ST) ^6^			**0.002**			0.346
Typhimurium ST313	0 (0.0)	49 (57.6)		6 (35.3)	54 (66.7)	
Typhimurium ST19	0 (0.0)	2 (2.4)		0 (0.0)	1 (1.2)	
Enteritidis ST11	1 (33.3)	33 (38.8)		5 (29.4)	24 (29.6)	
Enteritidis ST6	0 (0.0)	0 (0.0)		0 (0.0)	1 (1.2)	
Virchow ST16	2 (66.7)	1 (1.2)		1 (5.9)	0 (0.0)	
Heidelberg ST15	0 (0.0)	0 (0.0)		1 (5.9)	0 (0.0)	
Newport ST188	0 (0.0)	0 (0.0)		0 (0.0)	1 (1.2)	
Unknown ST1162	0 (0.0)	0 (0.0)		1 (5.9)	0 (0.0)	
Unknown ST1817	0 (0.0)	0 (0.0)		2 (11.8)	0 (0.0)	
Drug resistance						
TMP-SMX	5 (35.7)	98 (89.9)	**<0.001**	18 (34.0)	96 (91.4)	**<0.001**
Ampicillin	4 (28.6)	96 (88.9)	**<0.001**	20 (37.7)	94 (90.4)	**<0.001**
Chloramphenicol	3 (21.4)	91 (84.3)	**<0.001**	15 (28.3)	83 (79.0)	**<0.001**
Multi-drug resistant^7^	3 (21.4)	90 (83.3)	**<0.001**	14 (26.4)	81 (77.1)	**<0.001**

Numbers and percentages out of all non-missing records

^1^ P-value test for difference in the proportions / means of co-factors between NTS bacteremia and NTS diarrhea by age group, calculated using a chi-squared statistic for differences in proportions and T-test for differences in mean values. Fisher’s exact test used to test difference in proportions with cell values of 5 or less.

^2^ Among those with non-missing responses to having taken any medication for the current illness

^3^ HIV status ascertained through two community surveillance surveys conducted in 2008–2009 and 2013.

^4^Among those presenting with fever or history of fever

^5^Serogroup identified by commercial agglutinating antiserum

^6^Based on Multi-Locus Sequence Typing (MLST). Among 3 NTS diarrhea and 85 NTS bacteremia specimens genotyped from patients under age five; and among 16 NTS diarrhea and 81 NTS bacteremia specimens genotyped from patients age five and older.

^7^Resistance to TMP-SMX, Ampicillin, and Chloramphenicol

NTS bacteremia resulted predominantly from infection with *Salmonella* serogroups B and D (which include the most commonly occurring NTS serovars in Africa, *S*. Typhimurium *and S*. Enteritidis, respectively [16, 17, 19, 25–28]). In children under five, group B *Salmonella* was isolated from 64.1% of patients with bacteremia and 43.8% of patients with diarrhea (P < 0.001); and group D *Salmonella* was isolated from 34.2% of patients with bacteremia and 18.8% of patients with diarrhea (P < 0.001). A similar pattern was observed for individuals over age five. MDR was more common among NTS isolated from bacteremic patients compared to diarrheal patients in both those under 5 years (83.3% versus 21.4%, P <0.001) and those ≥ 5 years of age (77.1% versus 26.4%, P <0.001).

Multi-locus sequence typing (MLST) was conducted on a subsample of 185 stored NTS isolates of which 166 were from bacteremic patients and 19 were from diarrheal patients. Among eighty-five NTS isolates from patients under age five with bacteremia 57.6% were *S*. Typhimurium ST313, 38.8% were *S*. Enteritidis ST 11, and 1.2% were *S*. Virchow ST16. Among 81 NTS isolates genotyped from bacteremic patients over age five, 66.7% were *S*. Typhimurium ST313, 29.6% were *S*. Enteritidis ST11, 1.2% were *S*. Typhimurium ST19, 1.2% were *S*. Enteritidis ST6 and 1.2% were *S*. Newport ST188. Of three stool NTS samples from individuals under five years of age, one was *S*. Enteritidis ST11 and two were *S*. Virchow ST16. Of 16 stool NTS samples from individuals over age five, 6 (35.3%) were *S*. Typhimurium ST313 and 5 (29.4%) were *S*. Enteritidis ST11 (Table 1).

**Fig 2:**
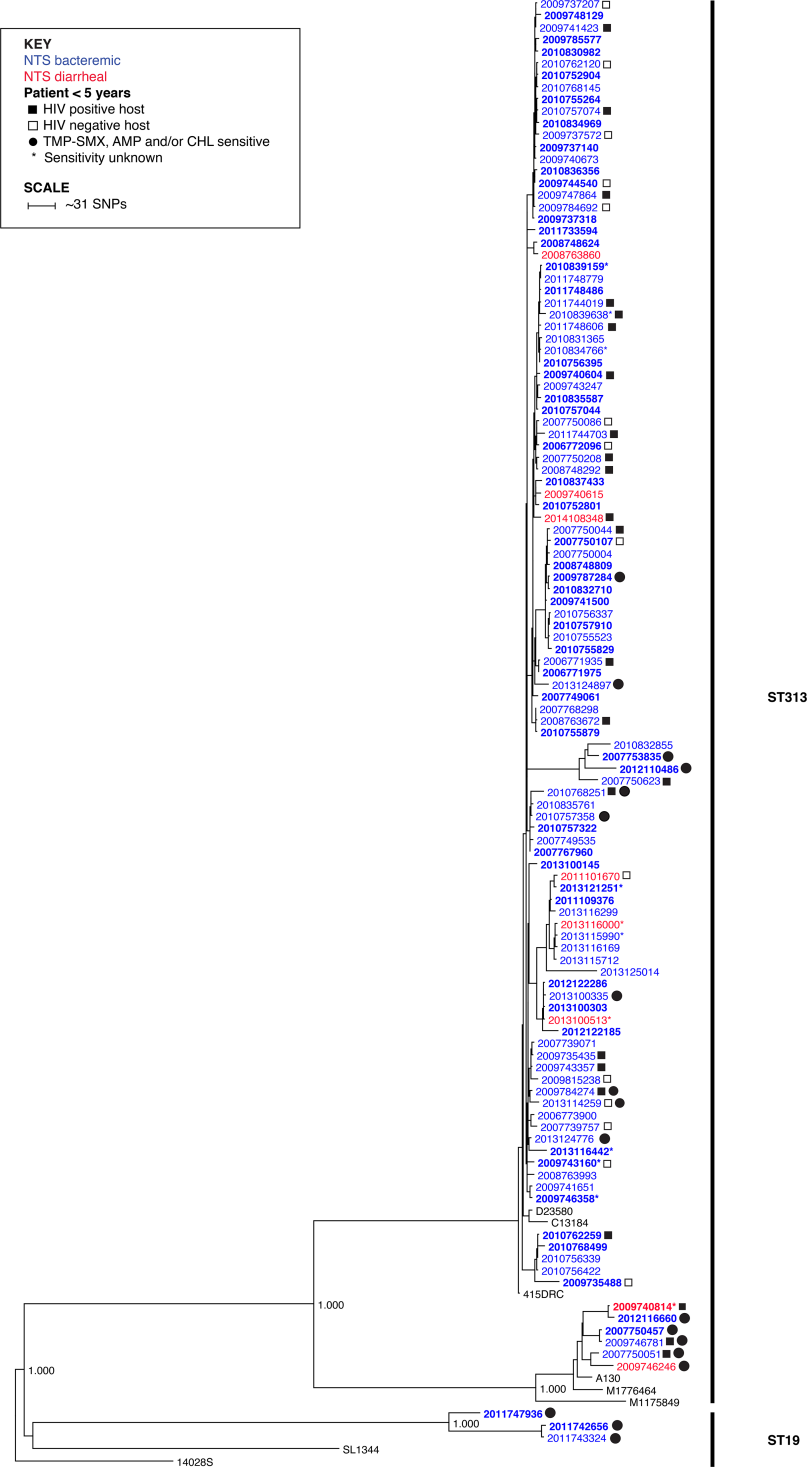
Maximum likelihood phylogeny of 115 S. Typhimurium strains (3 strains are from the same patient at separate visits within one month and are not included in the epidemiologic analysis) based on k-mers. Each strain is represented by collection year for clarity (strain numbers can be found in the S1 Table). NTS bacteremic and diarrheal clinical strains are colored blue and red, respectively. Reference strains appear in black text. Bold text indicates strains collected from patients under 5 years of age. Filled and open squares indicate HIV positive and negative hosts, respectively, when known. Clinical strains susceptible to TMP-SMX, ampicillin (AMP) and/or chloramphenicol (CHL) are indicated with filled circles. Strains with unknown susceptibilities are indicated with an asterisk (*). Basal branches with 100% local support are labeled. The tree was rooted with laboratory strain 14028S.

Phylogenetic reconstruction of 115 *S*. Typhimurium strains (106 from bacteremic patients, six from diarrheal patients, and three repeat tests from the same patient at separate visits within one month) grouped almost exclusively with the ST313 phylogenetic clade associated with strain D23580 (lineage 2) from Malawi [22] (Fig 2). Only nine *S*. Typhimurium strains grouped outside this clade: Six ST313 strains, including four bacteremic and two diarrheal strains, grouped with the chloramphenicol sensitive strain A130 from Malawi (lineage 1), which represents a ST313 lineage distinct from that of D23580 [16, 40]; and three strains that grouped outside the branch leading to the ST313 lineages were sequence type ST19 (Fig 2).

### Age-stratified analysis

Multivariate adjusted logistic regression models, stratified by individuals under and over age five were used to identify risk factors associated with NTS bacteremia compared to NTS diarrhea. Among children under age five, a total of 104 cases of NTS bacteremia and 10 cases of NTS diarrhea with non-missing data on age, year of diagnosis, concurrent malaria parasitemia, multi-drug resistance phenotype of the NTS isolate, and serogroup of the NTS isolate were included in the multivariate logistic regression model. (Table 2). MDR phenotypes were more common among isolates cultured from NTS bacteremic patients compared to NTS diarrhea patients, [aOR = 15.16, 95% CI (2.84–81.05), P = 0.001]. This association was independent of age, malaria, serogroup, and year of diagnosis. We observed a positive, though non-significant association between concurrent malaria parasitemia and NTS bacteremia. We found no evidence of an age association with NTS bacteremia versus diarrhea in children under age five.

**Table 2:** Association between host- and pathogen- characteristics and NTS clinical outcome (bacteremia vs. diarrhea), stratified on age under/over five years. Multivariate- adjusted odds ratios,indicate the odds of bacteremia divided by the odds of diarrhea by co-variate, adjusted for other co-variates. Note estimates not adjusted for HIV due to small sample size. See Table 3 for HIV-stratified results.

	Age < 5 years (N = 114)			Age ≥ 5 years (N = 116)		
	aOR	95% CI	P-value	aOR	95% CI	P-value
Age (years)	2.17	(0.48–9.84)	0.314	1.00	(0.97–1.03)	0.985
Year of diagnosis	1.39	(0.80–2.41)	0.245	1.24	(0.88–1.74)	0.226
Malaria (smear positive)	1.86	(0.23–14.72)	0.559	4.02	(0.43–37.28)	0.221
Serogroup						
Group B	1.97	(0.41–9.42)	0.394	0.44	(0.12–1.55)	0.202
*Salmonella* Spp	0.93	(0.05–17.14)	0.960	NA	-	-
Group D	1.00	ref	-	1.00	ref	-
Multi-drug resistant (MDR)	15.16	(2.84–81.05)	**0.001**	6.70	(2.25–19.89)	**0.001**

All cases of NTS serogroup C1/C2 were dropped from the multivariate model (N = 2 for age < 5 and N = 8) as they were only found in diarrhea patients and thus predicted NTS bacteremia versus diarrhea perfectly. Similarly, all cases of *Salmonella* spp. in individuals age five and older were dropped (N = 4) as they predicted NTS bacteremia versus diarrhea perfectly.

Among individuals age five and over, a total of 89 cases of NTS bacteremia and 27 NTS diarrhea cases were included in the model. Similar to children under age five, we found no evidence of an association between NTS bacteremia and age, concurrent malaria parasitemia, or serogroup in multivariate models (Table 2). MDR NTS was associated with NTS bacteremia as compared to diarrhea, [aOR = 6.70 95% CI (2.25–19.89), P = 0.001] in multivariate models. The association between MDR and NTS bacteremia, furthermore, held in secondary multi-variate analysis not adjusting for concurrent malaria, which enabled the inclusion of all individuals regardless of whether they were tested for malaria (n = 53 NTS diarrhea and 105 NTS bacteremic patients), [aOR = 7.07, 95% CI (2.89–17.33), P < 0.001].

### Serogroup-stratified analysis

Among the entire sample of individuals (including those both under and over age five), the association between MDR and NTS bacteremia was observed in those infected with *Salmonella* serogroup B, [aOR = 5.96, 95% CI (2.28–15.56), P < 0.001] and serogroup D, [aOR = 14.15, 95% CI (1.10–182.7), P = 0.042]. Serogroups C1/C2 were exclusively non-MDR and were only isolated from diarrheal patients.

### HIV-stratified analysis

The association between MDR phenotype and NTS bacteremia was strongest among HIV-uninfected individuals, [aOR = 22.61, 95% CI (2.53–185.0), P = 0.005], and was positive, but not significant among HIV-infected individuals [aOR = 2.74, 95% CI (0.48–15.66), P = 0.258] (Table 3). HIV infected individuals with either stool or blood NTS were more likely than HIV negative individuals to be infected with a MDR strain of NTS (72.0% versus 55.6%), [aOR = 2.97, 95% CI (1.11–7.95), P = 0.030].

**Table 3:** Multivariate-adjusted odds ratios stratified on HIV status.

Strata	HIV negative (n = 31)			HIV positive (n = 43)		
	aOR	95% CI	P-value	aOR	95% CI	P-value
Age (years)	1.00	(0.94–1.07)	0.942	1.00	(0.96–1.05)	0.950
Year of diagnosis	1.16	(0.47–2.90)	0.743	1.17	(0.42–3.24)	0.766
Serogroup						
Group B	0.55	(0.05–5.44)	0.607	0.61	(0.05–7.33)	0.697
Group D	1.00	Ref	-	1.00	Ref	-
Multi-drug resistant (MDR)	21.61	(2.53–184.99)	**0.005**	2.74	(0.48–15.66)	0.258

Concurrent malaria parasitemia was not found in any NTS diarrheal patients and was only found in one NTS bacteremia case and was therefore removed as a co-factor from the model

## Discussion

In this study we compared NTS bacteremic patients with NTS diarrhea patients and found strong evidence for an association between multi-drug resistant strains of NTS and invasiveness in the host [16, 17]. The association between MDR NTS was independent of age, NTS serogroup and persisted in HIV negative individuals. The low sample size of those with concurrent malaria parasitemia precluded a comprehensive analysis of the previously shown association between malaria parasitemia and invasive NTS. These findings suggest that in addition to known host-factors, pathogen-specific factors associated with multi-drug resistance may contribute significantly to NTS invasiveness.

The emergence of multi-drug resistant NTS in Africa has paralleled the increasing burden of invasive NTS [16, 17, 41, 42] and the biological mechanisms conferring increased virulence in drug resistant *Salmonella* strains have been proposed in novel clades of *S*. Typhimurium [22], and *S*. Enteritidis [43]. *S*. Typhimurium ST313 lineage 2 has multiple-linked antibiotic resistance determinants encoded by a virulence-associated plasmid associated with systemic disease [1, 16, 17, 44–46] and was recently introduced into Kenya [19]. Furthermore, a recently described African clade of multi-drug resistant *S*. Enteritidis sequence type 11, which has also been isolated from patients in Kenya [47], are genome degraded and putatively more invasive [43]. *S*. Enteritidis sequence type 11 was the second most common cause of invasive disease in our study, and MDR within that serotype was strongly associated with bacteremia. Further investigation of its genome repertoire may help confirm the role of genome degradation in invasive disease among *S*. Enteritidis.

This study adds to a growing body of evidence suggesting that *Salmonella* Typhimurium ST313 is a dominant genotype circulating in East Africa. Prior to this study, the occurrence of ST313 was described almost exclusively in young children and HIV positive individuals, and it is hypothesized that ST313 emerged as a result of its adaptation to the immune-compromised host [1, 16]. In our study, MDR ST313 represented more than half of all bloodstream NTS isolates among children under five and two thirds of all bloodstream NTS isolates among individuals over five, many of whom were HIV uninfected. Our results furthermore indicate that ST313 can be found in the stool of patients presenting with diarrhea. Among 16 diarrheal isolates that underwent full genome sequencing, 5 clustered with ST313 D23580.

We found that HIV was significantly more common among patients with NTS bacteremia compared to those with NTS diarrhea. Our results also suggest that HIV-positive individuals were disproportionately infected with MDR strains of NTS. Multiple previous studies have observed a large burden of NTS bacteremia among HIV-infected individuals [2, 5, 27], and the biological mechanisms by which HIV predisposes to disseminated infection are well established [1]. Aside from the immune-modulating mechanisms predisposing HIV-infected individuals to invasive infection from NTS, the pressure from prophylactic antibiotic use among HIV-infected individuals may select for infection with MDR strains. [48] [49, 50]. Still, we found that the association between MDR NTS and bacteremia was not explained entirely by HIV. In fact, we found strong evidence for an association between MDR NTS and bacteremia in HIV-uninfected individuals. We lacked power to show a significant association of MDR and NTS bacteremia in HIV-infected individuals, though a true association may have been masked by the already high risk of invasive salmonellosis in HIV-infected individuals regardless of MDR.

Our results must be considered in light of some limitations of the study design. There may be misclassification with respect to differentiating NTS diarrhea from NTS bacteremia, as well as misclassification with respect to HIV status. Secondly, while our study reports strong evidence for associations between MDR NTS and bacteremia, we were unable to evaluate the role of antibiotic treatment failure as a cause of more severe disease among those infected with resistant strains. In some settings, NTS strains resistant to more than one antimicrobial agent are associated with increased risk of severe clinical outcomes [51, 52], though this is not always the case [25]. More research is needed to differentiate pathogen-specific virulence from treatment failure in NTS clinical outcomes in Africa.

In summary, multi-drug resistant NTS was associated with bloodstream infection compared to diarrheal infection, independent of age and serogroup, and among HIV negative individuals. We found that *S*. Typhimurium sequence type ST313 is a common cause of NTS bacteremia, and to a lesser extent also causes NTS diarrhea. MDR is a major concern for the control of highly pathogenic NTS serotypes [53]. Whether MDR increases the risk of invasive disease because of virulence properties conferred at the genetic level or the association is a result of antibiotic treatment failure warrants further investigation.

### Sequence reads access

Sequence reads for all strains sequenced for this study have been submitted to the Short Read Archive (SRA) under BioProject PRJNA406975.

## Supporting information

S1 Table List of all non-typhoidal Salmonella isolates included in the primary analysis, listed in order by serogroup and sequence type. “Sequence type” and “Serovar” indicated for isolates that have been genotyped. Missing values indicate not tested or data unavailable.
(DOCX)

S2 Table Accession numbers for all *S*. Typhimurium isolates included in the phylogenetic tree. (XLSX)
