# Supplementary material for: Multi-drug resistant non-typhoidal Salmonella associated with invasive disease in western Kenya
Source: PLoS Negl Trop Dis. 2018 Jan 12;12(1):e0006156. doi: 10.1371/journal.pntd.0006156 (PMC5785031; doi:10.1371/journal.pntd.0006156)
Supplement: S1 Table — “Sequence type” and “Serovar” indicated for isolates that have been genotyped. Missing values indicate not tested or data unavailable. (DOCX) [file pntd.0006156.s001.docx]

**Supplementary table 1.** List of all non-typhoidal Salmonella isolates, included in the primary analysis. “Sequence type” and “Serovar” indicated for isolates that have been genotyped. Missing values indicate not tested or data unavailable.

|  |  |  |  |  | **Antibiotic susceptibility** | | |  |  |  |  |
| --- | --- | --- | --- | --- | --- | --- | --- | --- | --- | --- | --- |
| **Specimen ID** | **Sequence Type** | **Serovar** | **Serogroup** | **Clinical outcome** | **Chloramphenicol** | **Cotrimoxazole** | **Ampicillin** | **Date of isolation** | **Age (years)** | **HIV status** | **Malaria smear** |
| 2011749514 | 15 | Heidelberg | Salmonella Group B | diarrhea | susceptible | susceptible | susceptible | 11-Aug-11 | 36.2 | negative | negative |
| 2011743327 | 19 | Typhimurium | Salmonella Group B | bacteremia | susceptible | resistant | resistant | 4-Mar-11 | 28.4 |  |  |
| 2011747936 | 19 | Typhimurium | Salmonella Group B | bacteremia | susceptible | susceptible | susceptible | 15-Feb-11 | 0.8 |  | positive |
| 2011742656 | 19 | Typhimurium | Salmonella Group B | bacteremia | susceptible | susceptible | susceptible | 23-Aug-11 | 3.9 |  | positive |
| 2012101599 | 188 | Newport | Salmonella Group B | bacteremia | resistant | resistant | resistant | 15-Feb-12 | 49.5 | positive | negative |
| 2008763860 | 313 | Typhimurium | Salmonella Group B | diarrhea | resistant | resistant | resistant | 31-Oct-08 | 48.4 |  |  |
| 2011101670 | 313 | Typhimurium | Salmonella Group B | diarrhea | resistant | resistant | resistant | 18-Jul-12 | 12.9 | negative | negative |
| 2009740615 | 313 | Typhimurium | Salmonella Group B | diarrhea | resistant | resistant | resistant | 23-Mar-09 | 25.0 |  | negative |
| 2013116000 | 313 | Typhimurium | Salmonella Group B | diarrhea |  |  |  | 23-Sep-13 | 40.3 |  | negative |
| 2014108348 | 313 | Typhimurium | Salmonella Group B | diarrhea | resistant | resistant | resistant | 23-Apr-14 | 11.3 | positive |  |
| 2009746246 | 313 | Typhimurium | Salmonella Group B | diarrhea | susceptible | susceptible | resistant | 7-Mar-09 | 21.7 |  | negative |
| 2010755523 | 313 | Typhimurium | Salmonella Group B | bacteremia | resistant | resistant | resistant | 5-Apr-10 | 8.8 |  | positive |
| 2008763993 | 313 | Typhimurium | Salmonella Group B | bacteremia | resistant | resistant | resistant | 10-Nov-08 | 18.4 |  | negative |
| 2010839159 | 313 | Typhimurium | Salmonella Group B | bacteremia |  |  |  | 25-Jan-11 | 1.5 |  | negative |
| 2009743160 | 313 | Typhimurium | Salmonella Group B | bacteremia | resistant | resistant |  | 30-Jun-09 | 3.2 | negative | positive |
| 2007750208 | 313 | Typhimurium | Salmonella Group B | bacteremia | resistant | resistant | resistant | 14-Apr-08 | 46.8 | positive | negative |
| 2010762259 | 313 | Typhimurium | Salmonella Group B | bacteremia | resistant | resistant | resistant | 28-Apr-10 | 3.9 | positive | negative |
| 2006771935 | 313 | Typhimurium | Salmonella Group B | bacteremia | resistant | resistant | resistant | 4-Aug-08 | 10.0 | positive | negative |
| 2009787284 | 313 | Typhimurium | Salmonella Group B | bacteremia | susceptible | susceptible | susceptible | 4-Nov-09 | 1.1 |  | negative |
| 2010752904 | 313 | Typhimurium | Salmonella Group B | bacteremia | resistant | resistant | resistant | 2-Jun-10 | 3.3 |  | negative |
| 2013124776 | 313 | Typhimurium | Salmonella Group B | bacteremia | resistant | resistant | susceptible | 27-Dec-13 | 37.4 |  | negative |
| 2013116299 | 313 | Typhimurium | Salmonella Group B | bacteremia | resistant | resistant | resistant | 5-Oct-13 | 33.1 |  | negative |
| 2011744019 | 313 | Typhimurium | Salmonella Group B | bacteremia | resistant | resistant | resistant | 23-Apr-11 | 37.5 | positive | negative |
| 2009747864 | 313 | Typhimurium | Salmonella Group B | bacteremia | resistant | resistant | resistant | 27-Jul-09 | 41.3 | positive | negative |
| 2007750107 | 313 | Typhimurium | Salmonella Group B | bacteremia | resistant | resistant | resistant | 2-Apr-08 | 4.1 | negative | negative |
| 2013116442 | 313 | Typhimurium | Salmonella Group B | bacteremia |  |  |  | 14-Oct-13 | 4.0 |  | negative |
| 2013124897 | 313 | Typhimurium | Salmonella Group B | bacteremia | susceptible | resistant | resistant | 30-Dec-13 | 6.0 |  | negative |
| 2009746358 | 313 | Typhimurium | Salmonella Group B | bacteremia | resistant | resistant | resistant | 26-Jan-09 | 2.8 |  | positive |
| 2010768499 | 313 | Typhimurium | Salmonella Group B | bacteremia | resistant | resistant | resistant | 16-Jul-10 | 0.7 |  | negative |
| 2012122185 | 313 | Typhimurium | Salmonella Group B | bacteremia | resistant | resistant | resistant | 2-Jan-13 | 3.5 |  | positive |
| 2009748129 | 313 | Typhimurium | Salmonella Group B | bacteremia | resistant | resistant | resistant | 5-Aug-09 | 0.3 |  |  |
| 2010756422 | 313 | Typhimurium | Salmonella Group B | bacteremia | resistant | resistant | resistant | 22-Jun-10 | 28.2 | negative | negative |
| 2007750004 | 313 | Typhimurium | Salmonella Group B | bacteremia | resistant | resistant | resistant | 20-Mar-08 | 5.7 |  | negative |
| 2009735435 | 313 | Typhimurium | Salmonella Group B | bacteremia | resistant | resistant | resistant | 17-Nov-09 | 22.9 | positive | negative |
| 2009737572 | 313 | Typhimurium | Salmonella Group B | bacteremia | resistant | resistant | resistant | 10-Mar-10 | 11.6 | negative |  |
| 2008763672 | 313 | Typhimurium | Salmonella Group B | bacteremia | resistant | resistant | resistant | 16-Oct-08 | 39.3 | positive | negative |
| 2009737318 | 313 | Typhimurium | Salmonella Group B | bacteremia | resistant | resistant | resistant | 4-Mar-10 | 1.8 |  | positive |
| 2010755879 | 313 | Typhimurium | Salmonella Group B | bacteremia | resistant | resistant | resistant | 13-May-10 | 0.6 |  | negative |
| 2009744540 | 313 | Typhimurium | Salmonella Group B | bacteremia | resistant | resistant | resistant | 1-Jun-09 | 2.2 | negative | negative |
| 2010757358 | 313 | Typhimurium | Salmonella Group B | bacteremia | resistant | susceptible | susceptible | 2-Apr-10 | 38.8 |  | negative |
| 2009815238 | 313 | Typhimurium | Salmonella Group B | bacteremia | resistant | resistant | resistant | 29-Jan-10 | 64.7 | negative | negative |
| 2009741423 | 313 | Typhimurium | Salmonella Group B | bacteremia | resistant | resistant | resistant | 4-Dec-09 | 67.5 | positive | negative |
| 2010756339 | 313 | Typhimurium | Salmonella Group B | bacteremia | resistant | resistant | resistant | 18-Jun-10 | 8.6 |  | positive |
| 2010757322 | 313 | Typhimurium | Salmonella Group B | bacteremia | resistant | resistant | resistant | 1-Apr-10 | 1.9 |  | positive |
| 2010834969 | 313 | Typhimurium | Salmonella Group B | bacteremia | resistant | resistant | resistant | 30-Oct-10 | 1.4 |  | negative |
| 2009746781 | 313 | Typhimurium | Salmonella Group B | bacteremia | susceptible | resistant | resistant | 27-Feb-09 | 52.8 | positive | negative |
| 2010836356 | 313 | Typhimurium | Salmonella Group B | bacteremia | resistant | resistant | resistant | 11-Aug-10 | 0.6 |  | positive |
| 2013115712 | 313 | Typhimurium | Salmonella Group B | bacteremia | resistant | resistant | resistant | 12-Sep-13 | 7.3 |  |  |
| 2011748779 | 313 | Typhimurium | Salmonella Group B | bacteremia | resistant | resistant | resistant | 4-Apr-11 | 7.5 |  | positive |
| 2010752801 | 313 | Typhimurium | Salmonella Group B | bacteremia | resistant | resistant | resistant | 31-May-10 | 1.7 |  | negative |
| 2013100145 | 313 | Typhimurium | Salmonella Group B | bacteremia | resistant | resistant | resistant | 16-Jan-13 | 2.7 |  | negative |
| 2013100335 | 313 | Typhimurium | Salmonella Group B | bacteremia | susceptible | resistant | resistant | 21-Jan-13 | 36.4 |  |  |
| 2009741651 | 313 | Typhimurium | Salmonella Group B | bacteremia | resistant | resistant | resistant | 10-Dec-09 | 40.3 |  | positive |
| 2009743357 | 313 | Typhimurium | Salmonella Group B | bacteremia | resistant | resistant | resistant | 6-Jul-09 | 22.5 | positive | negative |
| 2010837433 | 313 | Typhimurium | Salmonella Group B | bacteremia | resistant | resistant | resistant | 22-Dec-10 | 3.6 |  |  |
| 2010768251 | 313 | Typhimurium | Salmonella Group B | bacteremia | susceptible | resistant | resistant | 12-Jul-10 | 30.1 | positive | negative |
| 2008748809 | 313 | Typhimurium | Salmonella Group B | bacteremia | resistant | resistant | resistant | 24-Jun-08 | 1.8 |  | negative |
| 2007768298 | 313 | Typhimurium | Salmonella Group B | bacteremia | resistant | resistant | resistant | 7-Oct-08 | 6.2 |  | positive |
| 2010768145 | 313 | Typhimurium | Salmonella Group B | bacteremia | resistant | resistant | resistant | 8-Jul-10 | 5.7 |  | positive |
| 2011748486 | 313 | Typhimurium | Salmonella Group B | bacteremia | resistant | resistant | resistant | 23-Mar-11 | 4.0 |  | negative |
| 2010755264 | 313 | Typhimurium | Salmonella Group B | bacteremia | resistant | resistant | resistant | 27-May-10 | 1.6 |  | negative |
| 2010756395 | 313 | Typhimurium | Salmonella Group B | bacteremia | resistant | resistant | resistant | 19-Jun-10 | 1.7 |  | negative |
| 2013125014 | 313 | Typhimurium | Salmonella Group B | bacteremia | resistant | resistant | resistant | 3-Jan-14 | 6.6 |  | positive |
| 2010762120 | 313 | Typhimurium | Salmonella Group B | bacteremia | resistant | resistant | resistant | 24-Apr-10 | 16.7 | negative | negative |
| 2008748292 | 313 | Typhimurium | Salmonella Group B | bacteremia | resistant | resistant | resistant | 16-May-08 | 46.9 | positive | negative |
| 2007750623 | 313 | Typhimurium | Salmonella Group B | bacteremia | resistant | resistant | resistant | 6-Jul-07 | 38.5 | positive | negative |
| 2007750086 | 313 | Typhimurium | Salmonella Group B | bacteremia | resistant | resistant | resistant | 31-Mar-08 | 7.1 | negative | negative |
| 2012116660 | 313 | Typhimurium | Salmonella Group B | bacteremia | susceptible | resistant | resistant | 15-Oct-12 | 2.3 |  | negative |
| 2009741500 | 313 | Typhimurium | Salmonella Group B | bacteremia | resistant | resistant | resistant | 7-Dec-09 | 3.9 |  | negative |
| 2008748624 | 313 | Typhimurium | Salmonella Group B | bacteremia | resistant | resistant | resistant | 10-Jun-08 | 1.5 |  | positive |
| 2007750457 | 313 | Typhimurium | Salmonella Group B | bacteremia | susceptible | resistant | resistant | 5-May-08 | 1.9 |  | negative |
| 2007750044 | 313 | Typhimurium | Salmonella Group B | bacteremia | resistant | resistant | resistant | 26-Mar-08 | 12.1 | positive | negative |
| 2010830982 | 313 | Typhimurium | Salmonella Group B | bacteremia | resistant | resistant | resistant | 3-Dec-10 | 0.7 |  | negative |
| 2009785577 | 313 | Typhimurium | Salmonella Group B | bacteremia | resistant | resistant | resistant | 13-Oct-09 | 1.7 |  | negative |
| 2006771975 | 313 | Typhimurium | Salmonella Group B | bacteremia | resistant | resistant | resistant | 7-Aug-08 | 2.2 |  | positive |
| 2007750051 | 313 | Typhimurium | Salmonella Group B | bacteremia | susceptible | resistant | resistant | 26-Mar-08 | 34.4 | positive | negative |
| 2010757074 | 313 | Typhimurium | Salmonella Group B | bacteremia | resistant | resistant | resistant | 26-Mar-10 | 41.8 | positive | negative |
| 2009740673 | 313 | Typhimurium | Salmonella Group B | bacteremia | resistant | resistant | resistant | 30-Mar-09 | 21.8 |  | negative |
| 2007749535 | 313 | Typhimurium | Salmonella Group B | bacteremia | resistant | resistant | resistant | 4-Feb-08 | 20.6 |  | negative |
| 2010839638 | 313 | Typhimurium | Salmonella Group B | bacteremia |  |  |  | 9-Feb-11 | 44.7 | positive | negative |
| 2010756337 | 313 | Typhimurium | Salmonella Group B | bacteremia | resistant | resistant | resistant | 18-Jun-10 | 16.3 |  | negative |
| 2009737207 | 313 | Typhimurium | Salmonella Group B | bacteremia | resistant | resistant | resistant | 2-Mar-10 | 11.7 | negative | positive |
| 2009784692 | 313 | Typhimurium | Salmonella Group B | bacteremia | resistant | resistant | resistant | 19-Aug-09 | 19.3 | negative | negative |
| 2010755829 | 313 | Typhimurium | Salmonella Group B | bacteremia | resistant | resistant | resistant | 12-May-10 | 2.9 |  | positive |
| 2010835587 | 313 | Typhimurium | Salmonella Group B | bacteremia | resistant | resistant | resistant | 15-Sep-10 | 0.6 |  | negative |
| 2007767960 | 313 | Typhimurium | Salmonella Group B | bacteremia | resistant | resistant | resistant | 10-Sep-08 | 0.8 |  | positive |
| 2013114259 | 313 | Typhimurium | Salmonella Group B | bacteremia | susceptible | resistant | susceptible | 1-Nov-13 | 15.9 | negative | positive |
| 2011744703 | 313 | Typhimurium | Salmonella Group B | bacteremia | resistant | resistant | resistant | 13-Jun-11 | 35.4 | positive |  |
| 2012110486 | 313 | Typhimurium | Salmonella Group B | bacteremia | susceptible | resistant | resistant | 28-Aug-12 | 1.6 |  | positive |
| 2010835761 | 313 | Typhimurium | Salmonella Group B | bacteremia | resistant | resistant | resistant | 29-Jul-10 | 5.6 |  | negative |
| 2007739757 | 313 | Typhimurium | Salmonella Group B | bacteremia | resistant | resistant | resistant | 14-Feb-07 | 6.1 | negative | negative |
| 2009784274 | 313 | Typhimurium | Salmonella Group B | bacteremia | susceptible | resistant | resistant | 6-Aug-09 | 53.1 | positive | negative |
| 2013100303 | 313 | Typhimurium | Salmonella Group B | bacteremia | resistant | resistant | resistant | 20-Jan-13 | 2.9 |  | positive |
| 2013121251 | 313 | Typhimurium | Salmonella Group B | bacteremia |  |  |  | 3-Dec-13 | 4.2 |  | negative |
| 2009740604 | 313 | Typhimurium | Salmonella Group B | bacteremia | resistant | resistant | resistant | 19-Mar-09 | 1.6 | positive | negative |
| 2006773900 | 313 | Typhimurium | Salmonella Group B | bacteremia | resistant | resistant | resistant | 22-Jan-07 | 54.6 |  | negative |
| 2011733594 | 313 | Typhimurium | Salmonella Group B | bacteremia | resistant | resistant | resistant | 4-Jul-11 | 3.1 |  | positive |
| 2007749061 | 313 | Typhimurium | Salmonella Group B | bacteremia | resistant | resistant | resistant | 3-Oct-07 | 1.2 |  | negative |
| 2009743247 | 313 | Typhimurium | Salmonella Group B | bacteremia | resistant | resistant | resistant | 2-Jul-09 | 28.5 |  | negative |
| 2009735488 | 313 | Typhimurium | Salmonella Group B | bacteremia | resistant | resistant | resistant | 18-Nov-09 | 2.9 | negative | negative |
| 2010832710 | 313 | Typhimurium | Salmonella Group B | bacteremia | resistant | resistant | resistant | 6-Nov-10 | 1.0 |  | negative |
| 2010831365 | 313 | Typhimurium | Salmonella Group B | bacteremia | resistant | resistant | resistant | 30-Sep-10 | 5.4 |  | positive |
| 2010757910 | 313 | Typhimurium | Salmonella Group B | bacteremia | resistant | resistant | resistant | 14-Apr-10 | 3.7 |  | positive |
| 2009737140 | 313 | Typhimurium | Salmonella Group B | bacteremia | resistant | resistant | resistant | 25-Feb-10 | 4.5 |  | negative |
| 2010832855 | 313 | Typhimurium | Salmonella Group B | bacteremia | resistant | resistant | resistant | 10-Nov-10 | 5.9 |  | negative |
| 2006772096 | 313 | Typhimurium | Salmonella Group B | bacteremia | resistant | resistant | resistant | 18-Aug-08 | 4.5 | negative | negative |
| 2011748606 | 313 | Typhimurium | Salmonella Group B | bacteremia | resistant | resistant | resistant | 28-Mar-11 | 29.3 | positive | negative |
| 2010757044 | 313 | Typhimurium | Salmonella Group B | bacteremia | resistant | resistant | resistant | 25-Mar-10 | 0.6 |  | negative |
| 2012122286 | 313 | Typhimurium | Salmonella Group B | bacteremia | resistant | resistant | resistant | 7-Jan-13 | 2.2 |  | positive |
| 2007739071 | 313 | Typhimurium | Salmonella Group B | bacteremia | resistant | resistant | resistant | 14-May-07 | 34.7 |  | negative |
| 2007753835 | 313 | Typhimurium | Salmonella Group B | bacteremia |  | resistant | susceptible | 10-Dec-07 | 2.8 |  | negative |
| 2013115990 | 313 | Typhimurium | Salmonella Group B | bacteremia |  |  |  | 23-Sep-13 | 40.3 |  | negative |
| 2013116169 | 313 | Typhimurium | Salmonella Group B | bacteremia | resistant | resistant | resistant | 7-Oct-13 | 15.2 |  | positive |
| 2011109376 | 313 | Typhimurium | Salmonella Group B | bacteremia | resistant | resistant | resistant | 14-Nov-11 | 1.5 |  | negative |
| 2011109222 | Indeterminate | Indeterminate | Salmonella Group B | diarrhea | susceptible | susceptible | susceptible | 8-Nov-11 | 50.4 | negative | negative |
| 2008748698 | Indeterminate | Indeterminate | Salmonella Group B | bacteremia | resistant | resistant | resistant | 16-Jun-08 | 35.0 |  | positive |
| 2009784599 | Indeterminate | Indeterminate | Salmonella Group B | bacteremia | resistant | resistant | resistant | 17-Aug-09 | 3.9 |  | negative |
| 2010762364 |  |  | Salmonella Group B | diarrhea | susceptible | resistant | resistant | 30-Apr-10 | 19.7 |  | negative |
| 2011743399 |  |  | Salmonella Group B | diarrhea | susceptible | susceptible | resistant | 7-Mar-11 | 30.4 |  | negative |
| 2009820625 |  |  | Salmonella Group B | diarrhea | susceptible | susceptible | susceptible | 15-Mar-10 | 33.7 | negative | negative |
| 2011733692 |  |  | Salmonella Group B | diarrhea | resistant | resistant | resistant | 6-Jul-11 | 36.1 | negative | negative |
| 2007750066 |  |  | Salmonella Group B | diarrhea | resistant | resistant | resistant | 26-Mar-08 | 12.1 | positive | negative |
| 2007739093 |  |  | Salmonella Group B | diarrhea | resistant | resistant | resistant | 16-May-07 | 30.9 | positive | negative |
| 2009820115 |  |  | Salmonella Group B | diarrhea | susceptible | susceptible | susceptible | 9-Jan-10 | 7.9 |  |  |
| 2010757796 |  |  | Salmonella Group B | diarrhea | susceptible | susceptible | susceptible | 12-Apr-10 | 59.8 | positive | negative |
| 2010757060 |  |  | Salmonella Group B | diarrhea | resistant | resistant | resistant | 25-Mar-10 | 0.6 |  | negative |
| 2007751849 |  |  | Salmonella Group B | diarrhea | susceptible | susceptible | susceptible | 19-Jun-07 | 35.6 | positive | negative |
| 2010833806 |  |  | Salmonella Group B | diarrhea | susceptible | resistant | resistant | 2-Jan-11 | 21.5 |  | negative |
| 2008748327 |  |  | Salmonella Group B | diarrhea | resistant | resistant | resistant | 20-May-08 | 45.9 |  | negative |
| 2009782821 |  |  | Salmonella Group B | diarrhea | susceptible | susceptible | susceptible | 9-Oct-09 | 1.0 |  | negative |
| 2011115507 |  |  | Salmonella Group B | diarrhea | resistant | resistant | resistant | 5-Jan-12 | 0.7 |  |  |
| 2007750589 |  |  | Salmonella Group B | diarrhea | resistant | resistant | resistant | 2-Jul-07 | 37.0 | positive | negative |
| 2008764068 |  |  | Salmonella Group B | diarrhea | resistant | resistant | resistant | 12-Nov-08 | 1.2 | negative | negative |
| 2007750912 |  |  | Salmonella Group B | diarrhea | resistant | resistant | resistant | 2-Jul-07 | 39.5 | positive |  |
| 2007739029 |  |  | Salmonella Group B | diarrhea | resistant | resistant | resistant | 9-May-07 | 22.1 |  | negative |
| 2007767976 |  |  | Salmonella Group B | diarrhea | susceptible | susceptible | susceptible | 12-Sep-08 | 1.2 |  | negative |
| 2009784639 |  |  | Salmonella Group B | diarrhea | susceptible | susceptible | susceptible | 18-Aug-09 | 16.7 | negative | negative |
| 2008761099 |  |  | Salmonella Group B | diarrhea | susceptible | susceptible | susceptible | 2-Dec-08 | 0.5 |  | positive |
| 2012109234 |  |  | Salmonella Group B | diarrhea | susceptible | susceptible | susceptible | 4-Feb-13 | 63.6 |  |  |
| 2009741010 |  |  | Salmonella Group B | diarrhea | susceptible | susceptible | susceptible | 4-May-09 | 14.4 | negative | negative |
| 2014108711 |  |  | Salmonella Group B | diarrhea | resistant | resistant | resistant | 12-Jun-14 | 42.2 |  | negative |
| 2011748767 |  |  | Salmonella Group B | diarrhea | resistant | resistant | resistant | 4-Apr-11 | 22.5 | positive | negative |
| 2007751043 |  |  | Salmonella Group B | diarrhea | susceptible | resistant | resistant | 16-Aug-07 | 1.5 |  |  |
| 2009743506 |  |  | Salmonella Group B | diarrhea | susceptible | susceptible | susceptible | 13-Jul-09 | 13.2 | negative | negative |
| 2009746805 |  |  | Salmonella Group B | bacteremia | resistant | resistant | resistant | 2-Mar-09 | 1.5 |  | positive |
| 2013101178 |  |  | Salmonella Group B | bacteremia | resistant | resistant | resistant | 8-Feb-13 | 5.3 |  |  |
| 2013124613 |  |  | Salmonella Group B | bacteremia |  |  |  | 13-Jan-14 | 66.6 |  | negative |
| 2014122863 |  |  | Salmonella Group B | bacteremia | resistant | resistant | resistant | 20-Sep-14 | 5.6 |  | positive |
| 2009747705 |  |  | Salmonella Group B | bacteremia | susceptible | susceptible | susceptible | 20-Jul-09 | 2.3 |  | positive |
| 2014122877 |  |  | Salmonella Group B | bacteremia | resistant | resistant | resistant | 21-Sep-14 | 77.3 |  | negative |
| 2014102550 |  |  | Salmonella Group B | bacteremia | resistant | resistant | resistant | 17-Mar-14 | 2.2 |  | negative |
| 2013100007 |  |  | Salmonella Group B | bacteremia | resistant | resistant | resistant | 14-Jan-13 | 1.2 |  | negative |
| 2010754879 |  |  | Salmonella Group B | bacteremia | resistant | resistant | resistant | 20-May-10 | 17.9 |  | positive |
| 2008748590 |  |  | Salmonella Group B | bacteremia | resistant | resistant | resistant | 8-Jun-08 | 22.4 |  | negative |
| 2009744519 |  |  | Salmonella Group B | bacteremia | susceptible | resistant | resistant | 28-May-09 | 30.7 |  | negative |
| 2009746817 |  |  | Salmonella Group B | bacteremia | susceptible | resistant | resistant | 3-Mar-09 | 4.5 |  | negative |
| 2009788446 |  |  | Salmonella Group B | bacteremia | susceptible | resistant | resistant | 10-Sep-09 | 30.9 |  |  |
| 2009821644 |  |  | Salmonella Group B | bacteremia | susceptible | resistant | resistant | 28-Dec-09 | 45.5 |  | negative |
| 2008761345 |  |  | Salmonella Group B | bacteremia | resistant | resistant | resistant | 16-Dec-08 | 3.3 |  | negative |
| 2010766055 |  |  | Salmonella Group B | bacteremia | resistant | resistant | resistant | 16-Mar-12 | 1.7 |  | negative |
| 2009744374 |  |  | Salmonella Group B | bacteremia | resistant | resistant | resistant | 18-May-09 | 1.5 |  | positive |
| 2014122869 |  |  | Salmonella Group B | bacteremia | resistant | resistant | resistant | 20-Sep-14 | 19.4 |  | negative |
| 2009740557 |  |  | Salmonella Group B | bacteremia | resistant | resistant | resistant | 16-Mar-09 | 23.1 | positive | negative |
| 2008764058 |  |  | Salmonella Group B | bacteremia | resistant | resistant | resistant | 13-Nov-08 | 2.4 | positive | negative |
| 2008761098 |  |  | Salmonella Group B | bacteremia | resistant | resistant | resistant | 2-Dec-08 | 1.7 |  | negative |
| 2009784868 |  |  | Salmonella Group B | bacteremia | resistant | resistant | resistant | 27-Aug-09 | 36.5 | positive | negative |
| 2011749457 |  |  | Salmonella Group B | bacteremia | resistant | resistant | resistant | 10-Aug-11 | 1.2 |  | positive |
| 2009782785 |  |  | Salmonella Group B | bacteremia | resistant | resistant | resistant | 9-Oct-09 | 10.3 |  | positive |
| 2008761028 |  |  | Salmonella Group B | bacteremia |  |  |  | 27-Nov-08 | 6.5 |  | positive |
| 2008763962 |  |  | Salmonella Group B | bacteremia | resistant | resistant | resistant | 6-Nov-08 | 1.6 | negative | positive |
| 2009747901 |  |  | Salmonella Group B | bacteremia | resistant | resistant | resistant | 28-Jul-09 | 32.2 | positive | negative |
| 2011109059 |  |  | Salmonella Group B | bacteremia | resistant | resistant | resistant | 2-Nov-11 | 2.3 |  | negative |
| 2008763989 |  |  | Salmonella Group B | bacteremia | resistant | resistant | resistant | 10-Oct-08 | 33.3 |  | negative |
| 2009788368 |  |  | Salmonella Group B | bacteremia | susceptible | resistant | resistant | 7-Sep-09 | 76.5 | negative |  |
| 2008761078 |  |  | Salmonella Group B | bacteremia | resistant | resistant | resistant | 1-Dec-08 | 2.4 |  | negative |
| 2010753178 |  |  | Salmonella Group B | bacteremia | resistant | resistant | resistant | 4-Feb-10 | 2.7 |  | positive |
| 2008761182 |  |  | Salmonella Group B | bacteremia | resistant | resistant | resistant | 8-Dec-08 | 1.0 |  | negative |
| 2009740798 |  |  | Salmonella Group B | bacteremia | susceptible | susceptible | susceptible | 13-Apr-09 | 2.6 | positive | negative |
| 2010832212 |  |  | Salmonella Group B | bacteremia |  |  |  | 23-Aug-10 | 32.0 | positive | negative |
| 2008761022 |  |  | Salmonella Group B | bacteremia | resistant | resistant | resistant | 27-Nov-08 | 1.6 |  | positive |
| 2008762848 |  |  | Salmonella Group B | bacteremia | susceptible | resistant | resistant | 22-Jan-09 | 1.3 |  | negative |
| 2009785682 |  |  | Salmonella Group B | bacteremia | resistant | resistant | resistant | 18-Oct-09 | 6.4 |  | negative |
| 2013115645 |  |  | Salmonella Group B | bacteremia | resistant | resistant | resistant | 7-Sep-13 | 0.6 |  | positive |
| 2009740699 |  |  | Salmonella Group B | bacteremia |  |  |  | 1-Apr-09 | 2.5 | positive | negative |
| 2009746532 |  |  | Salmonella Group B | bacteremia | susceptible | resistant | resistant | 10-Feb-09 | 1.1 |  | positive |
| 2008763964 |  |  | Salmonella Group B | bacteremia | resistant | resistant | resistant | 7-Nov-08 | 9.6 | positive | negative |
| 2014128882 |  |  | Salmonella Group B | bacteremia | resistant | resistant | resistant | 6-Dec-14 | 3.8 |  | negative |
| 2007753261 | 16 | Virchow | Salmonella Group C1/C2 | diarrhea |  |  |  | 7-Jul-08 | 1.0 |  | negative |
| 2014101462 | 16 | Virchow | Salmonella Group C1/C2 | diarrhea | susceptible | susceptible | resistant | 10-Feb-14 | 19.2 |  | negative |
| 2009821558 | 16 | Virchow | Salmonella Group C1/C2 | diarrhea | susceptible | susceptible | susceptible | 22-Dec-09 | 0.9 |  | negative |
| 2009788550 | 46 | Newport | Salmonella Group C1/C2 | diarrhea | susceptible | susceptible | susceptible | 14-Sep-09 | 20.8 | negative | negative |
| 2007750373 | 1817 | Indeterminate | Salmonella Group C1/C2 | diarrhea | susceptible | susceptible | susceptible | 25-Apr-08 | 75.9 |  | negative |
| 2012122081 | 1817 | Indeterminate | Salmonella Group C1/C2 | diarrhea | susceptible | susceptible | susceptible | 28-Dec-12 | 80.5 | negative |  |
| 2008763517 | Indeterminate | Indeterminate | Salmonella Group C1/C2 | diarrhea | susceptible | susceptible | susceptible | 6-Oct-08 | 30.3 | positive | negative |
| 2007750998 | Indeterminate | Indeterminate | Salmonella Group C1/C2 | diarrhea | susceptible | susceptible | susceptible | 13-Aug-07 | 57.7 | negative | positive |
| 2009744832 |  |  | Salmonella Group C1/C2 | diarrhea | susceptible | susceptible | susceptible | 15-Jun-09 | 53.0 | negative | negative |
| 2009821557 |  |  | Salmonella Group C1/C2 | diarrhea | susceptible | susceptible | susceptible | 21-Dec-09 | 5.2 |  |  |
| 2012112138 |  |  | Salmonella Group C1/C2 | diarrhea |  |  |  | 25-Sep-12 | 0.8 |  | negative |
| 2011735526 |  |  | Salmonella Group C1/C2 | diarrhea | susceptible | resistant | resistant | 4-Oct-11 | 41.3 |  | negative |
| 2014110631 |  |  | Salmonella Group C1/C2 | diarrhea |  |  |  | 23-Jun-14 | 35.7 |  |  |
| 2009788271 |  |  | Salmonella Group C1/C2 | diarrhea | susceptible | susceptible | susceptible | 2-Sep-09 | 5.6 |  |  |
| 2010832853 |  |  | Salmonella Group C1/C2 | diarrhea | susceptible | susceptible | susceptible | 9-Nov-10 | 53.5 |  |  |
| 2014124291 |  |  | Salmonella Group C1/C2 | diarrhea | susceptible | susceptible | susceptible | 23-Oct-14 | 13.6 |  | negative |
| 2009816373 |  |  | Salmonella Group C1/C2 | diarrhea | susceptible | susceptible | susceptible | 8-Feb-10 | 2.3 |  | positive |
| 2012111674 | 6 | Enteritidis | Salmonella Group D | bacteremia | resistant | resistant | resistant | 10-Sep-12 | 6.5 |  |  |
| 2012121385 | 11 | Enteritidis | Salmonella Group D | diarrhea | susceptible | susceptible | susceptible | 4-Dec-12 | 4.0 |  | positive |
| 2012121437 | 11 | Enteritidis | Salmonella Group D | diarrhea | susceptible | susceptible | susceptible | 5-Dec-12 | 13.8 |  |  |
| 2009746241 | 11 | Enteritidis | Salmonella Group D | diarrhea | resistant | susceptible | susceptible | 11-Mar-09 | 34.7 | positive |  |
| 2008763740 | 11 | Enteritidis | Salmonella Group D | diarrhea | susceptible | susceptible | susceptible | 22-Oct-08 | 45.4 | negative | negative |
| 2012121435 | 11 | Enteritidis | Salmonella Group D | diarrhea | susceptible | susceptible | susceptible | 5-Dec-12 | 30.5 |  |  |
| 2012121436 | 11 | Enteritidis | Salmonella Group D | diarrhea | susceptible | susceptible | susceptible | 5-Dec-12 | 9.1 |  |  |
| 2010839467 | 11 | Enteritidis | Salmonella Group D | bacteremia | resistant | resistant | resistant | 2-Feb-11 | 60.6 |  |  |
| 2012119109 | 11 | Enteritidis | Salmonella Group D | bacteremia | resistant | resistant | resistant | 21-Nov-12 | 2.1 |  | negative |
| 2010831864 | 11 | Enteritidis | Salmonella Group D | bacteremia | resistant | resistant | resistant | 13-Aug-10 | 1.3 |  | negative |
| 2009735450 | 11 | Enteritidis | Salmonella Group D | bacteremia | susceptible | susceptible | susceptible | 17-Nov-09 | 11.2 |  | negative |
| 2012112141 | 11 | Enteritidis | Salmonella Group D | bacteremia | susceptible | resistant | resistant | 21-Sep-12 | 38.5 |  | negative |
| 2010762162 | 11 | Enteritidis | Salmonella Group D | bacteremia | resistant | resistant | resistant | 26-Apr-10 | 3.1 | negative | negative |
| 2009816181 | 11 | Enteritidis | Salmonella Group D | bacteremia | resistant | resistant | resistant | 4-Feb-10 | 1.8 |  | negative |
| 2014100543 | 11 | Enteritidis | Salmonella Group D | bacteremia | susceptible | susceptible | resistant | 25-Jan-14 | 23.6 |  | negative |
| 2010767710 | 11 | Enteritidis | Salmonella Group D | bacteremia | resistant | resistant | resistant | 29-Jun-10 | 21.0 | negative | negative |
| 2013100184 | 11 | Enteritidis | Salmonella Group D | bacteremia | resistant | resistant | resistant | 16-Jan-13 | 3.4 |  | negative |
| 2009816517 | 11 | Enteritidis | Salmonella Group D | bacteremia | resistant | resistant | resistant | 9-Feb-10 | 23.4 |  |  |
| 2013112093 | 11 | Enteritidis | Salmonella Group D | bacteremia | resistant | resistant | resistant | 16-Jul-13 | 14.9 |  |  |
| 2013101765 | 11 | Enteritidis | Salmonella Group D | bacteremia | resistant | resistant | resistant | 21-Feb-13 | 1.7 |  |  |
| 2010830864 | 11 | Enteritidis | Salmonella Group D | bacteremia | resistant | resistant | resistant | 30-Nov-10 | 1.2 |  | negative |
| 2010768542 | 11 | Enteritidis | Salmonella Group D | bacteremia | resistant | resistant | resistant | 19-Jul-10 | 3.2 |  | negative |
| 2010832239 | 11 | Enteritidis | Salmonella Group D | bacteremia | resistant | resistant | resistant | 24-Aug-10 | 0.5 |  | negative |
| 2009746453 | 11 | Enteritidis | Salmonella Group D | bacteremia | resistant | resistant | resistant | 2-Feb-09 | 1.8 |  | negative |
| 2013101727 | 11 | Enteritidis | Salmonella Group D | bacteremia | resistant | resistant | resistant | 21-Feb-13 | 2.1 |  |  |
| 2010756500 | 11 | Enteritidis | Salmonella Group D | bacteremia | resistant | resistant | resistant | 21-Jun-10 | 4.2 |  | negative |
| 2012121921 | 11 | Enteritidis | Salmonella Group D | bacteremia | resistant | resistant | resistant | 21-Dec-12 | 1.4 |  | negative |
| 2011742853 | 11 | Enteritidis | Salmonella Group D | bacteremia | resistant | resistant | resistant | 2-Sep-11 | 1.0 |  | positive |
| 2011748279 | 11 | Enteritidis | Salmonella Group D | bacteremia | resistant | resistant | resistant | 24-Feb-11 | 36.9 |  | negative |
| 2010768712 | 11 | Enteritidis | Salmonella Group D | bacteremia | resistant | resistant | resistant | 21-Jul-10 | 4.8 |  | negative |
| 2013111294 | 11 | Enteritidis | Salmonella Group D | bacteremia |  |  |  | 4-Jul-13 | 2.8 |  | negative |
| 2010757964 | 11 | Enteritidis | Salmonella Group D | bacteremia | resistant | resistant | resistant | 14-Apr-10 | 21.9 | positive |  |
| 2012121360 | 11 | Enteritidis | Salmonella Group D | bacteremia | resistant | resistant | resistant | 3-Dec-12 | 1.5 |  | negative |
| 2010831928 | 11 | Enteritidis | Salmonella Group D | bacteremia | resistant | resistant | resistant | 16-Aug-10 | 6.1 |  | negative |
| 2010835888 | 11 | Enteritidis | Salmonella Group D | bacteremia | resistant | resistant | resistant | 30-Jul-10 | 1.8 |  | negative |
| 2013100687 | 11 | Enteritidis | Salmonella Group D | bacteremia | resistant | resistant | resistant | 28-Jan-13 | 1.4 |  | negative |
| 2010767948 | 11 | Enteritidis | Salmonella Group D | bacteremia | resistant | resistant | resistant | 5-Jul-10 | 7.9 |  | negative |
| 2010761858 | 11 | Enteritidis | Salmonella Group D | bacteremia | resistant | resistant | resistant | 20-Apr-10 | 26.6 | positive | negative |
| 2010754854 | 11 | Enteritidis | Salmonella Group D | bacteremia | resistant | resistant | resistant | 19-May-10 | 5.7 |  | negative |
| 2010768511 | 11 | Enteritidis | Salmonella Group D | bacteremia | resistant | resistant | resistant | 17-Jul-10 | 1.1 |  | negative |
| 2012109366 | 11 | Enteritidis | Salmonella Group D | bacteremia | resistant | resistant | resistant | 28-Jan-13 | 6.7 |  |  |
| 2010768080 | 11 | Enteritidis | Salmonella Group D | bacteremia | resistant | resistant | resistant | 7-Jul-10 | 3.4 |  | negative |
| 2012109546 | 11 | Enteritidis | Salmonella Group D | bacteremia | susceptible | susceptible | susceptible | 12-Aug-12 | 34.2 | negative | positive |
| 2013102904 | 11 | Enteritidis | Salmonella Group D | bacteremia | susceptible | susceptible | susceptible | 8-Apr-13 | 7.5 |  | negative |
| 2011743118 | 11 | Enteritidis | Salmonella Group D | bacteremia | resistant | resistant | resistant | 13-Sep-11 | 21.9 | positive | negative |
| 2012109593 | 11 | Enteritidis | Salmonella Group D | bacteremia | susceptible | susceptible | susceptible | 13-Aug-12 | 2.7 |  | negative |
| 2010757876 | 11 | Enteritidis | Salmonella Group D | bacteremia | resistant | resistant | resistant | 14-Apr-10 | 3.0 |  | positive |
| 2007739959 | 11 | Enteritidis | Salmonella Group D | bacteremia |  |  |  | 8-Mar-07 | 0.7 | positive | negative |
| 2010768609 | 11 | Enteritidis | Salmonella Group D | bacteremia | resistant | resistant | resistant | 19-Jul-10 | 29.9 | positive | negative |
| 2010761246 | 11 | Enteritidis | Salmonella Group D | bacteremia | susceptible | susceptible | susceptible | 28-Jun-10 | 2.5 |  | positive |
| 2012107395 | 11 | Enteritidis | Salmonella Group D | bacteremia | resistant | resistant | resistant | 26-Jun-12 | 3.8 |  | negative |
| 2010761806 | 11 | Enteritidis | Salmonella Group D | bacteremia | resistant | resistant | resistant | 17-Apr-10 | 23.3 | positive | positive |
| 2010757875 | 11 | Enteritidis | Salmonella Group D | bacteremia | resistant | resistant | resistant | 14-Apr-10 | 4.2 |  | positive |
| 2011735470 | 11 | Enteritidis | Salmonella Group D | bacteremia | resistant | resistant | resistant | 3-Oct-11 | 1.9 |  | positive |
| 2010756318 | 11 | Enteritidis | Salmonella Group D | bacteremia | resistant | resistant | resistant | 18-Jun-10 | 47.0 | negative | negative |
| 2011735668 | 11 | Enteritidis | Salmonella Group D | bacteremia | resistant | resistant | resistant | 13-Oct-11 | 3.0 |  | negative |
| 2011741443 | 11 | Enteritidis | Salmonella Group D | bacteremia | resistant | resistant | resistant | 29-May-12 | 29.0 |  | negative |
| 2010767855 | 11 | Enteritidis | Salmonella Group D | bacteremia | resistant | resistant | resistant | 2-Jul-10 | 4.0 |  | negative |
| 2013111316 | 11 | Enteritidis | Salmonella Group D | bacteremia | resistant | resistant | resistant | 4-Jul-13 | 2.7 |  | negative |
| 2007753464 | 11 | Enteritidis | Salmonella Group D | bacteremia | susceptible | susceptible | susceptible | 23-Jul-08 | 1.8 |  | negative |
| 2010836328 | 11 | Enteritidis | Salmonella Group D | bacteremia | susceptible | susceptible | susceptible | 11-Aug-10 | 13.8 |  | positive |
| 2010767725 | 11 | Enteritidis | Salmonella Group D | bacteremia | resistant | resistant | resistant | 29-Jun-10 | 1.4 |  | negative |
| 2013102292 | 11 | Enteritidis | Salmonella Group D | bacteremia | resistant | resistant | resistant | 7-Mar-13 | 5.4 |  | negative |
| 2010761235 | 11 | Enteritidis | Salmonella Group D | bacteremia | resistant | resistant | resistant | 27-Jun-10 | 25.0 | positive | negative |
| 2012121383 | Indeterminate | Indeterminate | Salmonella Group D | diarrhea | susceptible | susceptible | susceptible | 4-Dec-12 | 11.5 |  |  |
| 2014101900 | Indeterminate | Indeterminate | Salmonella Group D | bacteremia | resistant | resistant | resistant | 23-Feb-14 | 11.5 |  | negative |
| 2013114799 | Indeterminate | Indeterminate | Salmonella Group D | bacteremia | susceptible | susceptible | susceptible | 21-Nov-13 | 11.4 |  | negative |
| 2009816271 | Indeterminate | Indeterminate | Salmonella Group D | bacteremia | resistant | resistant | resistant | 5-Feb-10 | 4.4 |  | positive |
| 2013101874 | Indeterminate | Indeterminate | Salmonella Group D | bacteremia | resistant | resistant | resistant | 24-Feb-13 | 46.7 |  |  |
| 2007740214 |  |  | Salmonella Group D | diarrhea | susceptible | susceptible | susceptible | 4-Apr-07 | 24.3 | negative | negative |
| 2007750781 |  |  | Salmonella Group D | diarrhea |  |  |  | 23-Jul-07 | 45.4 |  |  |
| 2010757925 |  |  | Salmonella Group D | diarrhea | susceptible | susceptible | susceptible | 15-Apr-10 | 80.8 | negative | negative |
| 2009735555 |  |  | Salmonella Group D | diarrhea | susceptible | susceptible | susceptible | 5-Oct-09 | 2.2 |  | negative |
| 2010755459 |  |  | Salmonella Group D | diarrhea | susceptible | susceptible | susceptible | 4-May-10 | 5.1 |  | negative |
| 2009820502 |  |  | Salmonella Group D | diarrhea | resistant | resistant | resistant | 16-Jan-10 | 8.7 |  | positive |
| 2007748840 |  |  | Salmonella Group D | diarrhea | susceptible | resistant | susceptible | 17-Sep-07 | 1.5 |  | negative |
| 2014127312 |  |  | Salmonella Group D | bacteremia |  |  |  | 13-Nov-14 | 2.6 |  | negative |
| 2009785773 |  |  | Salmonella Group D | bacteremia | susceptible | susceptible | susceptible | 22-Oct-09 | 36.4 |  | negative |
| 2012117129 |  |  | Salmonella Group D | bacteremia |  |  |  | 30-Oct-12 | 2.4 |  | negative |
| 2013123851 |  |  | Salmonella Group D | bacteremia | susceptible | susceptible | susceptible | 18-Dec-13 | 12.6 |  | negative |
| 2009744332 |  |  | Salmonella Group D | bacteremia |  |  |  | 12-May-09 | 30.9 | positive | negative |
| 2014127340 |  |  | Salmonella Group D | bacteremia | susceptible | resistant |  | 13-Nov-14 | 71.4 |  | negative |
| 2011737252 |  |  | Salmonella Group D | bacteremia | susceptible | susceptible | susceptible | 9-May-11 | 0.8 |  | positive |
| 2011737666 |  |  | Salmonella Group D | bacteremia | resistant | resistant | resistant | 18-May-11 | 52.9 | positive |  |
| 2011748009 |  |  | Salmonella Group D | bacteremia | resistant | resistant | resistant | 17-Feb-11 | 0.7 |  | negative |
| 2010832316 |  |  | Salmonella Group D | bacteremia | resistant | resistant | resistant | 26-Aug-10 | 2.3 |  | negative |
| 2012101584 |  |  | Salmonella Group D | bacteremia |  |  |  | 14-Feb-12 | 31.1 |  | negative |
| 2009788335 |  |  | Salmonella Group D | bacteremia | resistant | resistant | resistant | 7-Sep-09 | 1.4 |  | negative |
| 2009816201 |  |  | Salmonella Group D | bacteremia | resistant | resistant | resistant | 4-Feb-10 | 11.7 |  | negative |
| 2013115575 | 16 | Virchow | Salmonella Spp | bacteremia | susceptible | susceptible | susceptible | 4-Sep-13 | 1.0 |  | negative |
| 2013112809 | 1162 | Indeterminate | Salmonella Spp | diarrhea | susceptible | susceptible | susceptible | 22-Jul-13 | 37.0 |  | negative |
| 2007751081 | Indeterminate | Indeterminate | Salmonella Spp | diarrhea | susceptible | susceptible | susceptible | 20-Aug-07 | 8.6 |  | positive |
| 2012105384 | Indeterminate | Indeterminate | Salmonella Spp | diarrhea | susceptible | susceptible | susceptible | 19-Jun-12 | 0.7 |  | negative |
| 2014124070 |  |  | Salmonella Spp | diarrhea |  |  |  | 15-Oct-14 | 21.8 |  | negative |
| 2009737465 |  |  | Salmonella Spp | diarrhea | susceptible | susceptible | susceptible | 8-Mar-10 | 0.8 |  | negative |
| 2007749258 |  |  | Salmonella Spp | diarrhea | susceptible | resistant | susceptible | 18-Oct-07 | 49.3 | positive | negative |
| 2010839315 |  |  | Salmonella Spp | diarrhea | susceptible | susceptible | susceptible | 28-Jan-11 | 80.6 | negative | negative |
| 2009787084 |  |  | Salmonella Spp | bacteremia | susceptible | susceptible | susceptible | 30-Oct-09 | 0.6 |  | negative |
| 2014102813 |  |  |  | bacteremia |  |  |  | 24-Mar-14 | 65.8 |  | negative |
